# Sex-biased cooperation among immature peers: it matters who helps whom

**DOI:** 10.1098/rspb.2025.1891

**Published:** 2025-11-26

**Authors:** Abhay Gupta, Carita Lindstedt, Nina Gerber, Raphael Ritter, Hanna Kokko

**Affiliations:** ^1^ Biological Sciences, Indian Institute of Science Education and Research Mohali, Manauli, Punjab, India; ^2^ Institute of Organismic and Molecular Evolution, Johannes Gutenberg University Mainz, Mainz, Rhineland-Palatinate, Germany; ^3^ Institute for Quantitative and Computational Biosciences, Johannes Gutenberg University Mainz, Mainz, Rhineland-Palatinate, Germany; ^4^ Department of Forest Sciences, University of Helsinki, Helsinki, Uusimaa, Finland; ^5^ Foundation Kora, 3063 Ittigen, Switzerland

**Keywords:** public good, anti-predator defence, evolution of cooperation, sex-biased helping, juvenile cooperation, haplodiploidy

## Abstract

Our understanding of sex-biased helping has progressed from a historical emphasis on relatedness differences caused by haplodiploidy to an understanding of the role played by the rarer-sex effect. Theory to date typically assumes that offspring help their mother. We show that an alternative, peer-to-peer cooperation can shed light on the interaction of helpers and the recipients of help. In pine sawfly (*Diprion pini*) larvae, larval peer-to-peer cooperation takes the form of collective, and individually costly, anti-predator behaviour. Larvae typically occur in mixed-sex groups, but females can lay unfertilized eggs that develop into haploid males, which produce male-only broods. Female-biased sex ratios typically select for female-biased helping, and our model here matches empirical findings. Alternative scenarios provide insight too: (i) if genetic constraints permit no sex-specificity in behaviour beyond haploid males expressing all alleles while helping in females can be recessive or dominant, then the sex difference in helping simply reflects the effects of dominance and (ii) female-biased helping can also emerge under male-biased sex ratios, if males are mostly produced in single-sex broods by unmated mothers. While this last example remains hypothetical for *D. pini*, it highlights an underappreciated point: the rarer-sex effect impacts solutions not only by modifying fitness prospects of the helper, but also of the recipient of help.

## 1. Introduction

The evolution of cooperation is a classic conundrum in evolutionary biology, where much theory has been devoted to understanding its emergence among individuals that are in the same ‘state’ (peer-to-peer cooperation *sensu* [1]) or where, following a common asymmetry in cooperative breeding vertebrates as well as in eusocial insects, offspring help their parent(s) in brood rearing or related tasks. In both contexts, sex biases in helping are commonplace (though not universal [2–4]). Most dramatic examples are in Hymenoptera, where the worker caste is exclusively female.

Hamilton [5,6] famously considered haplodiploidy to predispose female offspring to become helpers as a result of each female being more related to her sister than to her own offspring. This implies that a female offspring benefits more from helping her mother produce full siblings than being a reproductive herself. However, since the siblings thus produced can be either male or female, and a worker female is more related to her own son than to a son produced by her mother, the modern view is that Hamilton’s argument was incomplete. When considering sons as well as daughters, one has to additionally remember that the value of offspring is elevated due to the rarer-sex effect if sex ratios are biased [7]. Additionally, sex ratio effects [7] can be usefully combined with a consideration of likely ancestral states, where one of the sexes may be preadapted to help. For example, if helping requires brood care adaptations and mothers were ancestrally the only caring sex, it is easier to evolve female helpers than male helpers [8,9], while if colony defence is the main task of helpers and neither sex is preadapted to soldiering, then one expects unbiased helping with respect to sex [8].

Other asymmetries may, too, favour helping by a particular sex: in vertebrates, one sex may disperse more for a multitude of reasons [10] and selection typically favours less helping in that sex ([4,11]; see also [12] for a related argument, though without two sexes). Simultaneously, sex ratio effects remain important in vertebrates too. Male-biased sex ratios are associated with males being helpers [13,14]. Ecological factors such as the difference between temperate and tropical climates also covary with the sex of helpers, and this has been argued to operate via a cool climate (low adult survival) associating with a male surplus who can then be predisposed to helping [3].

Interestingly, theoretical work on sex-biased helping has largely adopted the assumption structure where offspring help their parents ([7,9], but see [11] for the possibility that dispersers help or harm after settling in a new location). Since the flow of help from offspring to parents is a hallmark of eusocial societies and typical cooperative breeders, this particular modelling set-up of past work is entirely justifiable (it is a common assumption structure in models of social behaviour as a whole [1]). However, this also constrains the models to not shed light on the recipient of help: it is simply assumed to be the mother (or, in [11], other adult breeders). Here, we highlight a case where peer-to-peer cooperation occurs among larvae of a haplodiploid insect, which allows us to investigate the roles of haplodiploidy, rarer-sex effects and the sex of the helper as well as the recipient of help in a uniquely separable manner. Interestingly, the peer-to-peer context creates same-sex and mixed-sex helping contexts, and we show this to matter such that overall sex ratio effects and ploidy differences are not the only causes of sex biases in helping.

**Figure 1.**
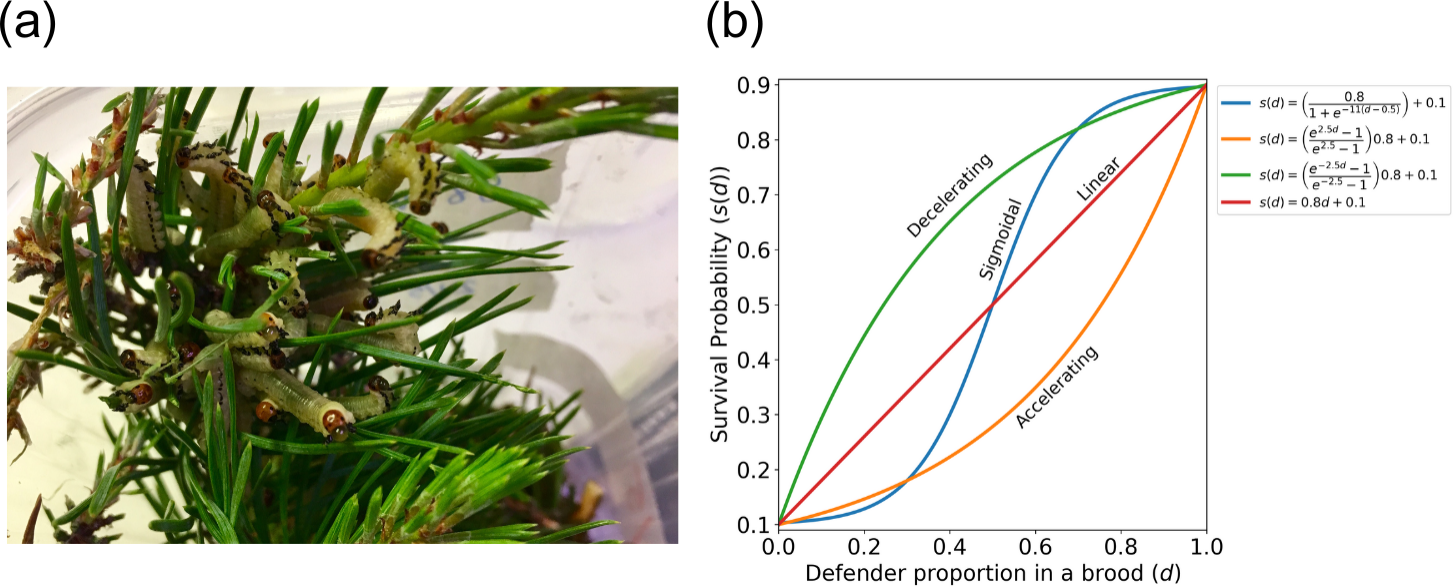
(a) A group of pine sawfly larvae adopting the defensive position. (b) Model assumptions relate the proportion of phenotypic defenders in the brood, d, to the survival of larvae in the brood, $s\left(d\right)$, with four possible shapes that our examples are based on. We present results based on the sigmoidal curve in the main text and the complete set in the electronic supplementary material.

Our model is inspired by chemically defended *Diprion pini* pine sawflies (Hymenoptera, Diprionidae). Upon perceiving a predator attack, larvae of this species (figure 1a) use a chemical defence strategy to synchronously regurgitate a resinous fluid, which deters predators [15,16]. The system satisfies the structure of a potentially altruistic trait [17]. Chemical defence in general has been shown to be a common good, where a higher frequency of defending individuals and/or close vicinity to defending individuals can improve the survival of a group (evidence across multiple species: [18–20]). In pine sawflies, producing the fluid and then losing it repetitively is costly, decreasing performance measures such as growth and immune defence, but also deflating individuals’ ability to defend in future encounters with predators [16,21,22]. Pine sawfly larvae differ in their willingness to deploy the fluid, with a general female bias: females are more likely to deploy these fluids, as well as producing a higher quantity [22]. Individuals that fail to produce any fluid when provoked appear to ‘free-ride’: they grow faster, i.e. cheat [22].

Interestingly, female-biased sex ratios are common in sawfly populations across different sawfly species [23], and *D. pini* is no exception (see electronic supplementary material, figure S1). Since the ability to deploy the fluid is not sex dependent (i.e. all individuals sequester and fill their defensive containers with the fluid), but the ‘willingness’ to engage in defence (i.e. deploy and lose the fluid) appears to be, we explore here how defence dynamics evolve, assuming that defence contributes to the common good of the larval group surviving to pupation, but is individually (and potentially sex-specifically) costly.

## 2. Models

We present two model variants for our main model (presented here in the main text), while extending the analysis to coevolutionary scenarios between sex ratios and defence effort in the electronic supplementary material.

In both variants of our main model, we assume that each individual expresses a single locus with two alleles, denoted 0 and 1, where 1 stands for defending and 0 for non-defending. In the first, *constrained* model, both sexes express the same locus. We thus assume no sex-specific evolution is possible beyond the fact that females are diploid (and thus we need to make some assumptions about dominance) while males are haploid. We consider the evolution of defence, assuming that, in females, the defence behaviour is either a dominant (model 1D) or a recessive (model 1R) trait. In the second, *unconstrained* model, we assume the opposite and make males and females maximally able to evolve different behaviours. We do this with the aid of a two-locus model: one locus is expressed in haploid males, while the other is expressed in females, once again with defence being either dominant (model 2D) or recessive (model 2R). Finally, we also provide a supplementary model that considers more continuous variation as well as coevolution between sex ratios and cooperation.

We assume that larvae occur in groups produced by a single female [17,24]. Because sawflies are haplodiploid, they are able to create male offspring without having mated [23]. Broods of unmated females consist of males only (electronic supplementary material, figure S1, [23]), and any helping that occurs in these groups occurs solely in a within-sex setting of males. Mated females produce a sex ratio that depends on the ratio of unfertilized to fertilized eggs. We do not make an assumption that this ratio is necessarily optimized (except in an additional model presented in electronic supplementary material, S7), as maximal insights into the effects of sex ratio can be gained by considering the consequences of all sex ratios, from heavily male-biased to heavily female-biased. We thus consider that a brood is produced without mating with a probability u (thus a proportion u of broods are male-only), and in the remaining broods (proportion 1-u), the sex ratio is r sons to 1-r daughters (see table 1 for all variable names). The population-wide sex ratio of offspring is thus $u+\left(1-u\right)r$. Inheritance follows normal Mendelian rules with haplodiploidy, i.e. sons inherit one of the mother’s alleles (one locus in model R, two loci in model depending on the model), while daughters inherit one allele from each of their two parents. We assume no linkage, though this is unimportant in the present setting where each individual only expresses one of the (maximally) two loci and mating is random.

**Table 1.** A brief description of all the parameters. A more comprehensive list of variables and parameters can be found in the supplementary material (electronic supplementary material, table S1).

variable	definition
B≥0	number of eggs laid by non-defender females (integer).
$0\leq c_{f}\leq 1$	cost to defending females; values are such that $\left(1-c_{f}\right)B$ is an integer.
$0\leq c_{m}\leq 1$	cost to defending males; values can range anywhere between 0 and 1 since this is applied as reduced mating success for defending males.
0≤u≤1	proportion of eggs produced asexually, i.e. by unmated females.
0≤r≤1	sex ratio of males in sexually produced broods.
0≤d≤1	proportion of defenders in a brood.
$0\leq s\left(d\right)\leq 1$	probability of survival of brood based on proportion of defenders, d.

Regardless of having been produced with or without mating, each offspring survives to maturity with a probability $s\left(d\right)$, where d is the proportion of defenders in the brood that the larva belongs to. Note that the own defence effort does not improve the survival of self beyond the effect that it contributes to $s\left(d\right)$ (the common good). As $s\left(d\right)$ denotes survival over presumably many attacks by different predators during larval growth, it is *a priori* difficult to predict its shape [18]; we use four different shapes (figure 1) to cover a wide range of possibilities. We present results using the sigmoidal shape in the main text and note that qualitatively, any of the other choices produce similar conclusions (electronic supplementary material, figures S2–S7 present the whole set).

Survivors are collected into a pool of adults. Adult fitness depends on whether the individual is phenotypically a defender: survivors may be non-defenders (free-riders) or defenders, but the latter paid costs for their contributions to the common good.

We assume a multiplicative cost. For females, we assume that phenotypic defenders lay fewer eggs: $\left(1-c_{f}\right)B$ eggs (in a single clutch), where $0\leq c_{f}\leq 1$ denotes the female cost. Phenotypic non-defenders lay B eggs.

Males, likewise, suffer a multiplicative cost $c_{m}$, but as males do not lay eggs, this is interpreted as reduced mating success. Apart from the reduced mating success of defender males (see electronic supplementary material for the exact implementation), mating is random.

We iterate the deterministic dynamics of discrete generations, tracking allele frequencies until equilibrium is reached (for details, see electronic supplementary material) and examine the evolved sex-specific frequencies of individuals with the defender phenotype, as well as the overall defence rate, computed as the weighted mean of the sex-specific phenotypes, with the offspring sex ratio determining the weighting. Since males are haploid, the phenotype and genotype frequencies are the same; for females, the phenotype also depends on dominance and differs between model versions R (defending is assumed to be recessive) and D (defending is dominant).

## 3. Results

### (a) Constrained model: costs determine overall contributions to the common good, but dominance determines the sex bias of helping

**Figure 2.**
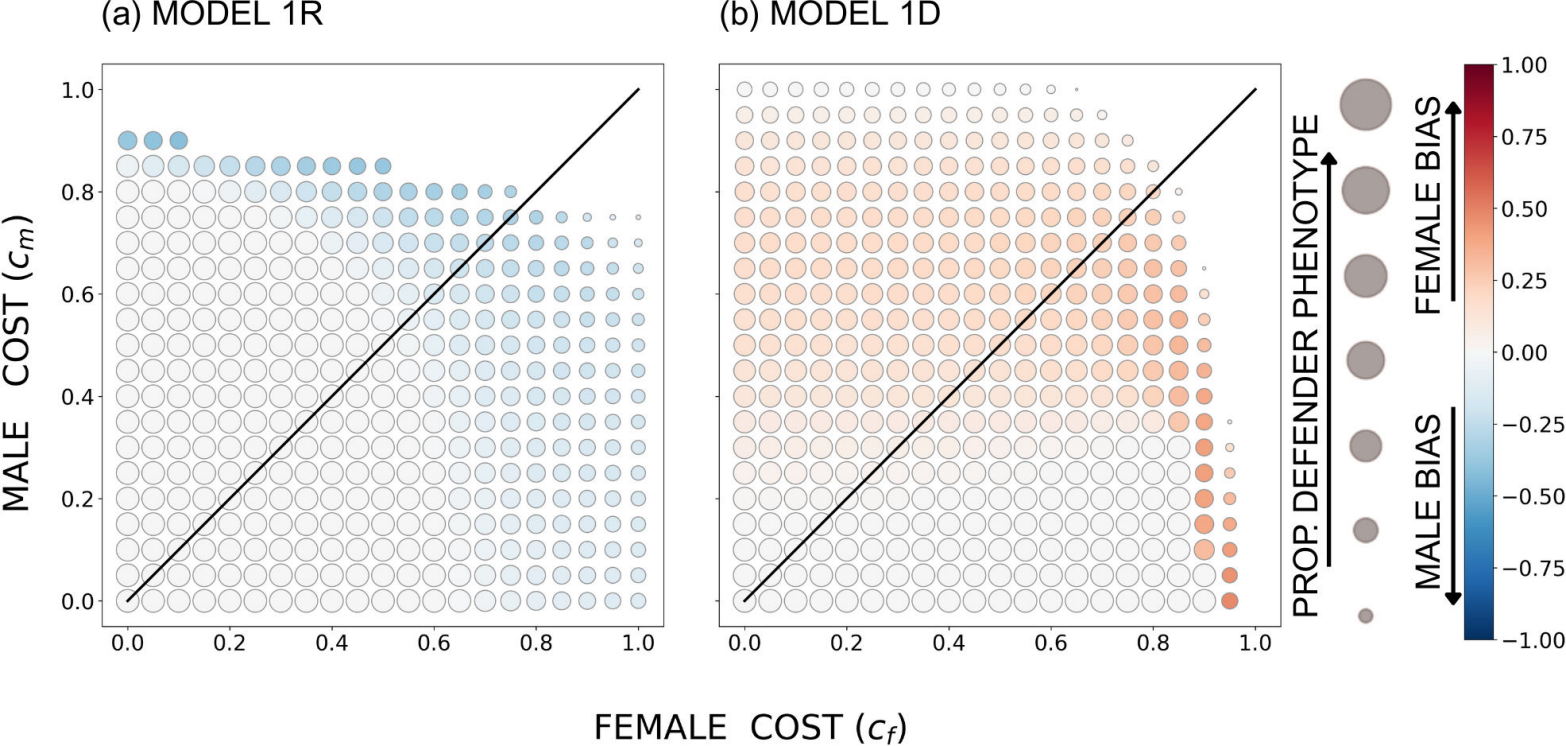
The equilibrium outcome for the constrained model with u=0.05, r=0.4737 (such that population-wide sex ratio among larvae is 0.5), B=20 and a sigmoidal survival function *s*(*d*); in (a) the defender allele is recessive and in (b) it is dominant. The size of the circles indicates the overall proportion of the defender phenotype, and the colour shade indicates female or male bias (the between-sex difference in the proportion of phenotypic defenders), with red denoting females contributing more (per capita) to the common good and blue denoting males contributing more. The diagonal indicates equal costs to males and females.

We consider the constrained model first. Unsurprisingly, the strongest contributions to the public good (hereafter ‘helping’) are found when the costs for both sexes are low (largest circles are always near the origin where $c_{f}=c_{m}=0$, figure 2). If every individual helps (allele 1 is fixed), there can also be no sex bias in helping, whether it is recessive or dominant in females: thus near $c_{f}=c_{m}=0$, all circles are white, indicating no sex bias.

The situation changes at higher costs. The overall proportion of defenders decreases (smaller circles, figure 2), and sex differences start playing a role. In the constrained model, recessivity always predicts male-biased helping (blue shades, figure 2), while dominance predicts a female bias (red shades, figure 2; the finding remains unchanged in a population where one sex is being overproduced, electronic supplementary material, figure S2).

Details of figure 2 may appear counterintuitive at first, because the sex bias is clearest when the costs of helping are absent in one sex, and in this case, the sex that helps more is the cost-suffering sex (i.e. darkest blue in figure 2 when the female cost is 0, and the darkest red region when the male cost is 0). However, recall that in the constrained model, the sexes are not able to adjust their helping independently, but simply express the consequences of an overall proportion for the frequency of an allele. If offspring hatch with a proportion x for the helping allele, and mating is random, then males have the helping allele phenotype with probability x while females will help with a probability of $x^{2}$ in the recessive case but with $x^{2}+2x\left(1-x\right)$ in the dominant case; the ratio of female to male help is thus $x^{2}/x=x$ in the recessive case, and $\left(x^{2}+2x\left(1-x\right)\right)/x=2-x$ in the dominant case. The former is smaller than 1 if helping is not fixed (x<1), i.e. recessivity forces females to help less. Under the same conditions, x<1, the latter value, 2-x, exceeds 1, i.e. dominance gives females no other option than to help more than males.

### (b) Unconstrained model: rarer-sex effects matter not only with respect to helper sex, but also with respect to the recipient of help

**Figure 3.**
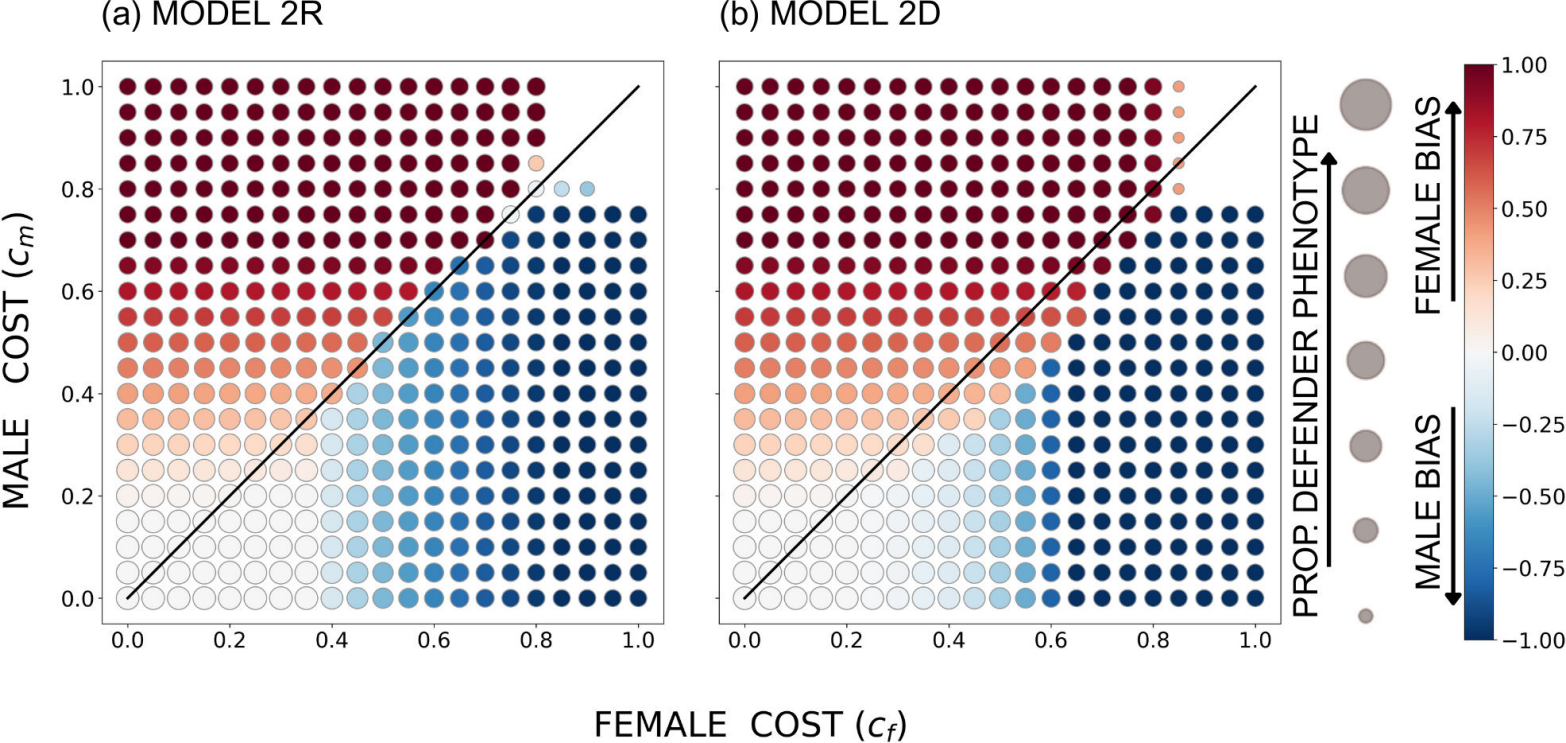
The equilibrium outcome for the unconstrained model with u=0.05, r=0.4737 (such that population-wide sex ratio among larvae is 0.5), B=20 and a sigmoidal survival function *s*(*d*); in (a) the defender allele is recessive and in (b) it is dominant. The size of the circles indicates the overall proportion of the defender phenotype, and the colour shade indicates female or male bias (the between-sex difference in the proportion of phenotypic defenders), with red denoting females contributing more (per capita) to the common good and blue denoting males contributing more. The diagonal indicates equal costs to males and females.

**Figure 4.**
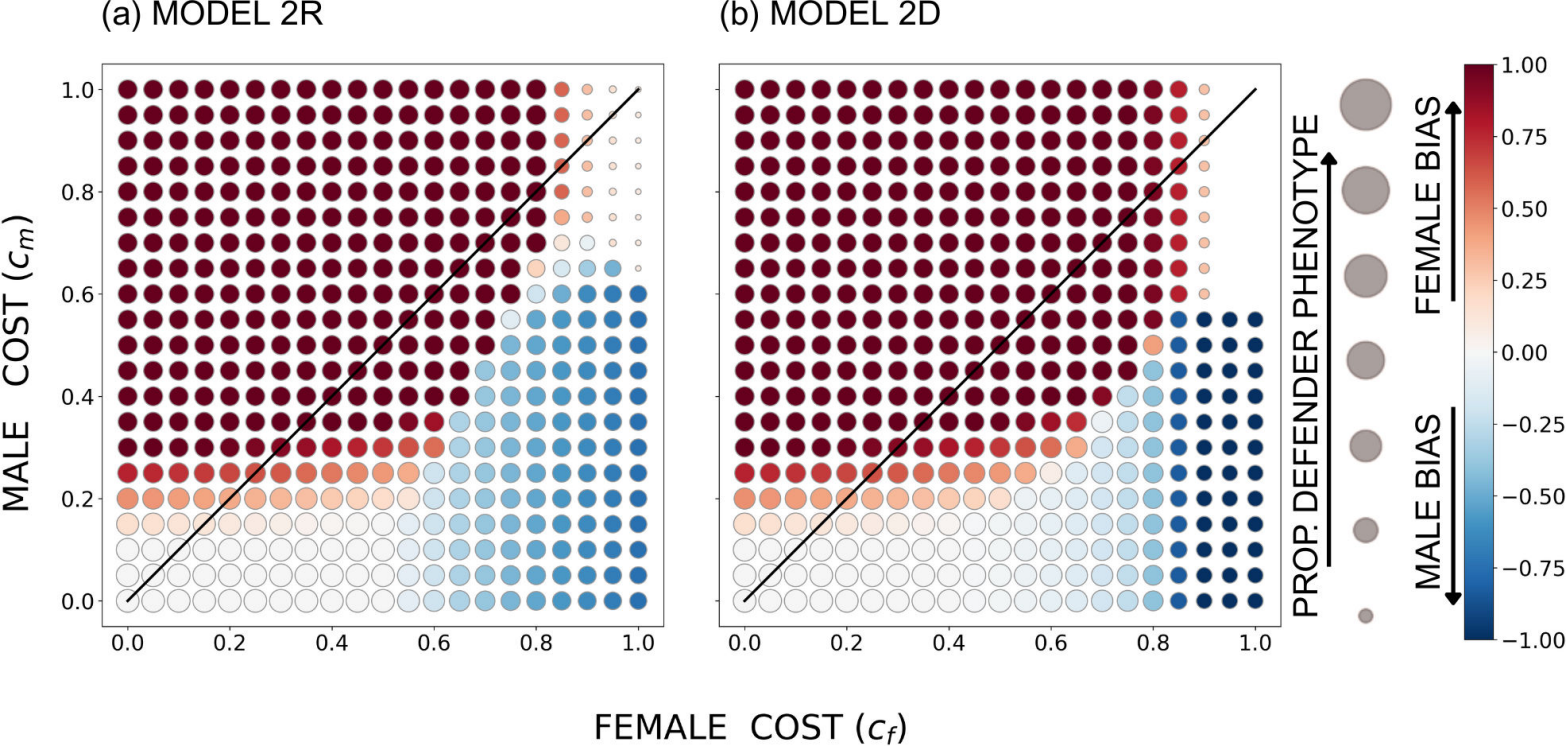
The equilibrium outcome for the unconstrained model with u=0.05, r=0.3 (such that the population-wide sex ratio among larvae is female-biased, with $u+\left(1-u\right)r=0.335$ as the proportion of males), B=20 and a sigmoidal survival function *s*(*d*); in (a) the defender allele is recessive and in (b) it is dominant. The size of the circles indicates the overall proportion of the defender phenotype, and the colour shade indicates female or male bias (the between-sex difference in the proportion of phenotypic defenders), with red denoting females contributing more (per capita) to the common good and blue denoting males contributing more. The diagonal indicates equal costs to males and females.

**Figure 5.**
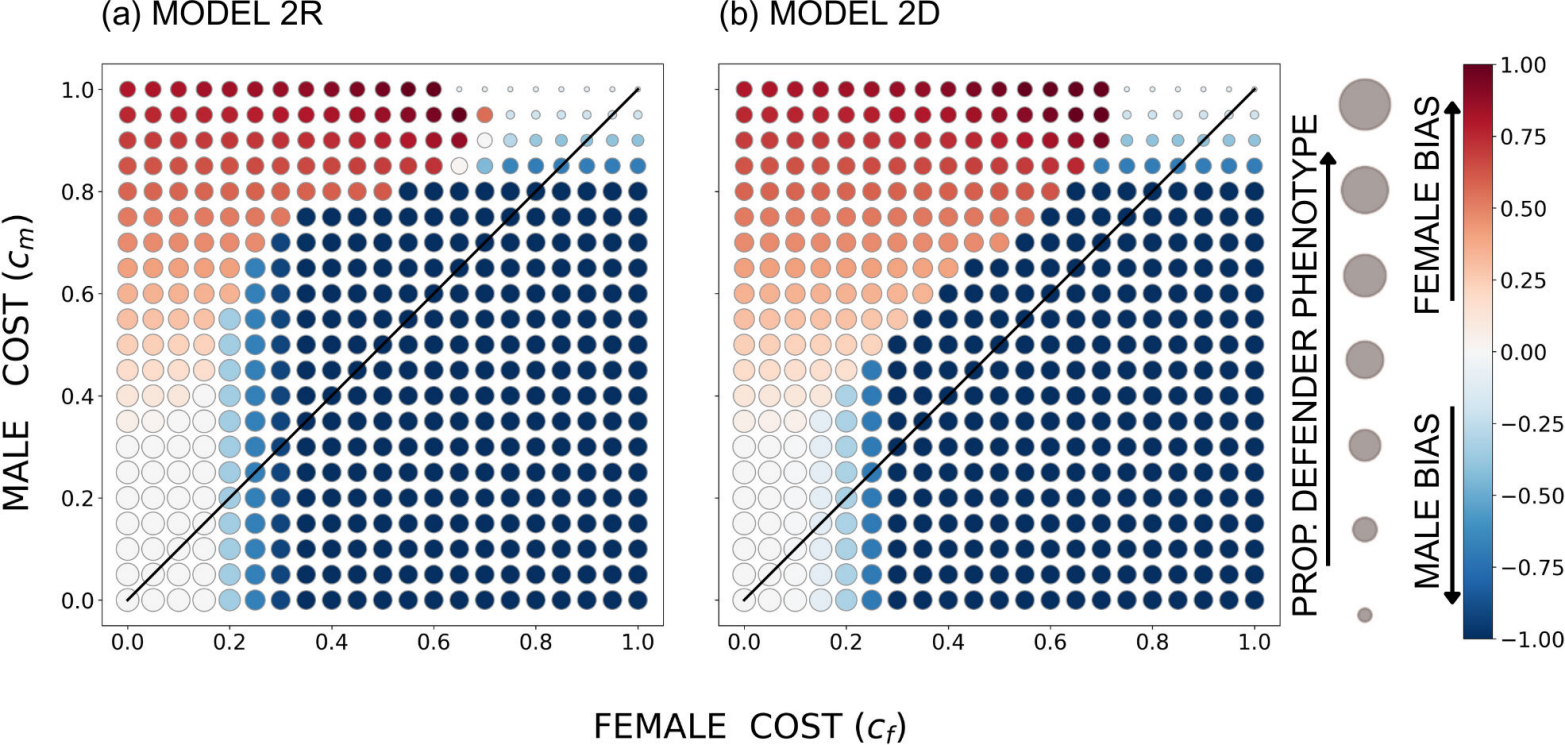
The equilibrium outcome for the unconstrained model with u=0.05, r=0.6474 (such that the population-wide sex ratio among larvae is male-biased, with $u+\left(1-u\right)r=0.665$ as the proportion of males), B=20 and a sigmoidal survival function *s*(*d*); in (a) the defender allele is recessive and in (b) it is dominant. The size of the circles indicates the overall proportion of the defender phenotype, and the colour shade indicates female or male bias (the between-sex difference in the proportion of phenotypic defenders), with red denoting females contributing more (per capita) to the common good and blue denoting males contributing more. The diagonal indicates equal costs to males and females.

When the genetic architecture allows the two sexes to evolve their own levels of help (but with intertwined fitness consequences as the two sexes mix their contributions to the common good), the costs behave intuitively. The overall pattern is that a cost asymmetry aligns with a helping asymmetry, with a high male cost $c_{m}$ creating female-biased helping and vice versa (figures 3–5) irrespective of whether female helping is a recessive or a dominant trait. An exception arises when helping costs are small enough relative to the benefit, so that all individuals evolve to contribute maximally (white circles in figures 3–5). The region where this happens is largest in the ecological setting where achieving good survival $s\left(d\right)$ requires that most individuals contribute (compare e.g. figure 3b with the other cases in figure 3).

However, if sex-specific costs were the only factor at play, one would expect the sex bias in helping (colours in figures 3–5) to be separated by the diagonal where $c_{f}=c_{m}$. Instead, we find females helping to extend to a parameter region where $c_{f}>c_{m}$ (i.e. females help more despite suffering stronger costs) when the larval population is female-biased as a whole (figure 4)—within reason, i.e. as long as the female cost does not massively exceed the male cost. Likewise, male helping extends to the parameter region where $c_{m}>c_{f}$ when the larval population is male-biased (figure 5).

This is clearly the rarer-sex effect at work. The common sex has poorer future prospects, and this tips the balance towards helping by that sex. It is important for this effect that in mixed-sex broods, at least some of the recipients of help are of the opposite, i.e. rare sex, and the common sex can gain inclusive fitness by helping the entire brood survive, including members of the valuable rare sex.

The above contains a caveat: the rarer-sex effect is based on some recipients of help being particularly worthwhile, but for this to work to promote helping by the opposite (common) sex, the sexes have to co-occur in mixed broods. Since male sawfly larvae can also be produced by unmated females, there is the potential for males to sometimes occur in male-only larval groups (in our model, a proportion u of broods are produced in this way). In this case, the above logic, where rarer-sex individuals get help by common-sex individuals, is broken: there are no individuals with asymmetric prospects within the same brood.

**Figure 6.**
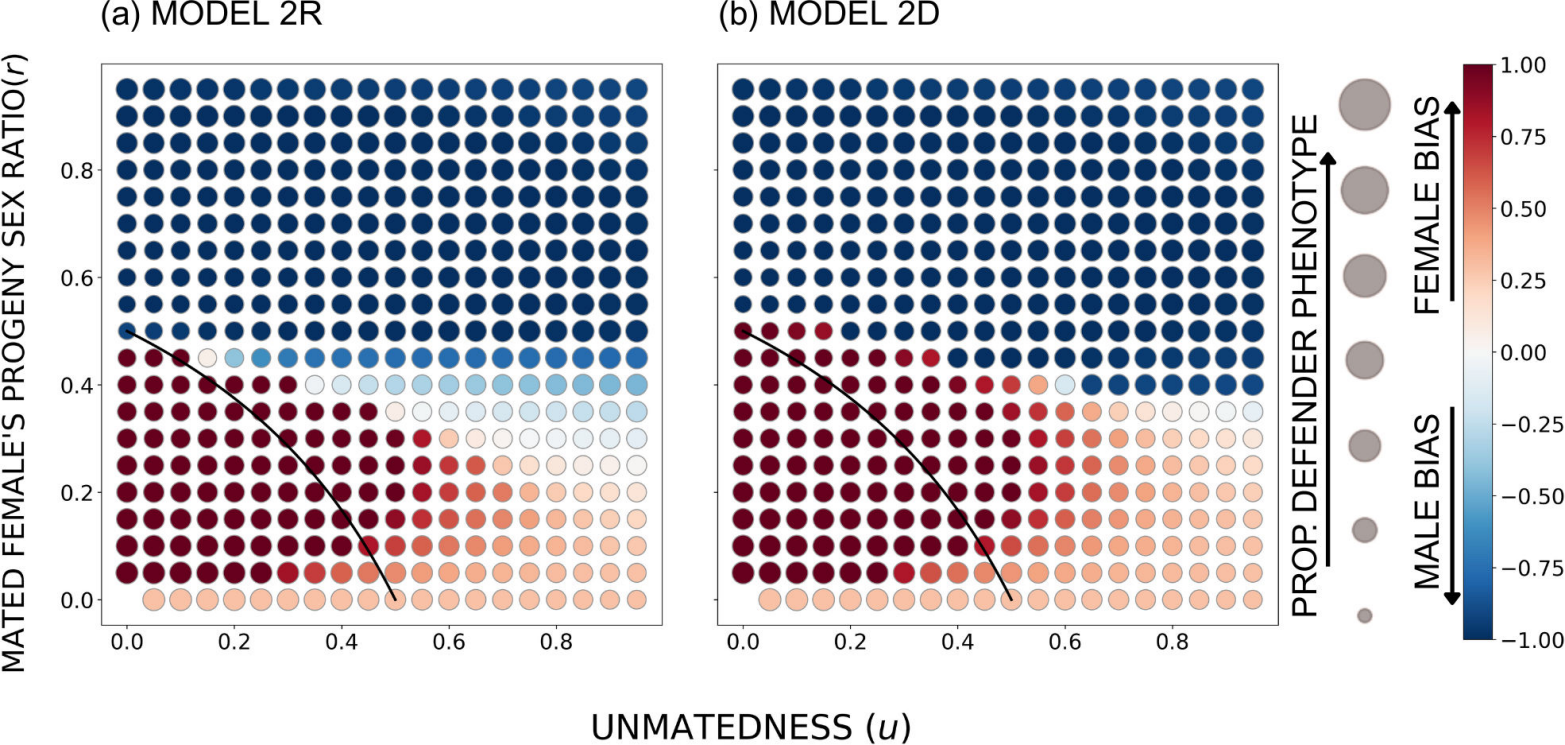
The equilibrium outcome for the unconstrained model while varying the population-wide sex ratio (using u and r) with $c_{f}=c_{m}=0.7$, B=20 and a sigmoidal survival function *s*(*d*); in (a) the defender allele is recessive and in (b) it is dominant. The size of the circles indicates the overall proportion of the defender phenotype, and the colour shade indicates female or male bias (the between-sex difference in the proportion of phenotypic defenders), with red denoting females contributing more (per capita) to the common good and blue denoting males contributing more. The black curve indicates the points where population-wide sex ratio among larvae is 0.5. Below the curve, the sex ratio is female-biased; above it, it is male-biased.

Consequently, the population-wide larval sex ratio $u+\left(1-u\right)r$ does not capture all effects of sex ratio; the situation is different if many males are produced via u (unmatedness) being high, or via r (sex ratio among the progeny of mated females) being high. This expectation is also borne out by the model when modifying the sex ratio systematically, with u and r varying independently of each other (figure 6). Here, the sex ratio is female-biased near the origin (below the black curve), and male-biased elsewhere. When females are the rarer sex because u is high, female-biased helping can prevail; but this ceases to be true when females are the rarer sex because r is high.

The reason is that high u creates male-only broods (see electronic supplementary material, figure S7), which has multiple consequences. The givers and recipients of help now have no asymmetry: males now mostly co-occur with other males (electronic supplementary material, figure S7), and if they have low reproductive value (recall that females create some of their offspring without mating, which lowers the value of males, [25]), the male-only broods mean that individuals with low prospects can only help others with equally poor prospects. The lack of asymmetry, where poor prospect individuals could help others with better prospects, hinders helping by males. The hindrance is milder when males are primarily produced because *r* is high (electronic supplementary material, figure S7), and here, male-biased helping can evolve (figure 6). Note also that sex biases in defence effort remain mild in the special case of *r* = 0 and *u* > 0 (lowest dots in figure 6): in this special (biologically unlikely) case, the task of surviving is performed in wholly sexually segregated groups. Here, effectively, each sex is given the same task to solve, with similar levels of effort expected as a whole.

The above insights, from both the constrained and the unconstrained model, are based on an ‘open’ model approach *sensu* [26], meaning that we report results for any combination of parameter values (particularly *u* and *r*) to disentangle their effects on the evolution of defence. This has the advantage of clarifying the causalities, but there are also advantages to a ‘closed’ approach [26], where one additionally takes into account that only some combinations of *u* and *r* are likely to occur in nature. For example, real sawfly populations are female-biased, and some of the above insights will not play out in this species. Thus, we built a supplementary model that restricts the view to the most likely parameter combinations by considering how unmatedness *u* and sex ratio (produced by mated mothers) *r* coevolve. The ‘closed’ model (electronic supplementary material) is able to predict female-biased sex ratios. Females evolve to be the more helpful sex in the closed model (electronic supplementary material, figure S9) as they do, at female-biased sex ratios, in the main model too (figure 6, cases below the black curve). This also matches reality [22].

## 4. Discussion

Our theoretical developments offer an explanation for female-biased helping in pine sawflies: female-biased sex ratios, as observed in nature, can make it profitable for the more common sex to help the rarer sex survive. Simultaneously, our models have more general messages, as the unique features of this system allow to tease apart effects of helper sex from those of the sex of the recipient of help.

Specifically, we show that both helper sex and recipient sex play a role in the rarer-sex effect. The former has already been identified to be important for the evolution of sex-biased helping, in the sense that an individual with poor reproductive prospects (a member of the common sex) has less to lose if it forgoes its own reproduction [4,7,11–14]. Here, we highlight that the rarer-sex effect also operates on the recipient side. If individuals occur in mixed-sex groups where help is both given and received, such that the locally produced common good (collective helping) can be improved by individuals with poor prospects (common sex) and at least some of the beneficiaries are of the rare sex, then we predict a clear sex bias with the common sex being the main contributor to the common good (all else being equal, its own reproductive value is poorer). If, on the other hand, a common sex often has to cooperate on its own, the opposite sex being absent (e.g. if haploid male groups are often produced by unmated females, and if males as a whole are the common sex), our models predict the common sex to be the less helpful sex despite its own poor reproductive value. Males in this context simply lack a route that allows channelling benefits to individuals with high reproductive value.

In other respects, our results show similar trends as earlier work, particularly that of [9], who showed that the ability to help is a major determinant of evolved patterns. While our model does not have an explicit term for helping ability, the cost of helping (paid in the adult stage) can be sex-specific, and high helping ability can be considered analogous to low cost paid when helping. Our results confirm that sex-specific costs have the expected effect on the outcome, but only when the behaviours of the two sexes are not strongly impacted by genetic constraints. If they are, then the effects of genetic architecture completely take over: haploid males contribute more than diploid females if contributing to the common good is a recessive trait (in the diploid sex), and less if it is a dominant trait.

Like all models, ours comes with some simplifications. We assumed defence to be entirely genetic, while in reality, sawfly larvae grow in groups that may experience changes in group composition over time. Phenotypic plasticity could thus have an impact on willingness to engage in group defence, and plastic responses could occur in response to the number of individuals in a group as well as their behaviour [17]. For simplicity, we assumed that the proportion of defenders (d) is relevant for group survival, not the absolute size of the group. One reason why this may matter is that defending females were assumed to be less fecund as adults, thus their broods will, all else being equal, have fewer larvae contributing to the brood-specific common good. We do not, however, believe that making survival depend on absolute brood size would undermine the basic messages of our model. While this statement would require modelling to verify it, it is based on intuition drawn from the current model results, which proved very robust to changes in the shape of the survival function (we considered four very different shapes, always with the same qualitative results regarding the interaction of sex-specific costs, the rarer-sex effect and genetic architecture).

That said, it is interesting to reflect on potential sex-specificities of life histories in more detail. In insects, selection for higher fertility, i.e. larger size in females, may in the present context create a pre-adaptation for female-biased helping, if allometry allows a large individual to secrete a sufficient volume of defence fluid with, effectively, a smaller cost paid. Empirically, the selfish benefit of cheating, i.e. non-defending, appears to have similar effects on both sexes in terms of growth, but this relationship was found to be significant only for males [22].

As a whole, our work shows that the effects of sex ratio are not solely captured by the low reproductive value of the more common sex; who the individuals end up interacting with also matters [1]. While sawflies are haplodiploid, there are ample examples of non-haplodiploid situations where the group identities of helpers and recipients of help differ in some sense, including the sexual subgroupings that our model considers. For example, intragroup coalitions are a common feature of mammalian societies. Recent work [27] expressed some surprise that there is much diversity: coalitions may consist of both sexes, but a large (33%) proportion of cases feature male-only coalitions, followed by 18% of female-only coalitions. The broad search of [27] did not reveal strong clues as to when to expect each style of coalition.

Our study helps to make sense of this diversity. Diploid mammals have, on average, equal reproductive value among males and females. This creates an expectation of diversity in the type of coalitions that form, in the following sense. When the baseline for cooperative behaviour is *a priori* similar across the sexes, small differences in sex-specific routes to fitness are sufficient to tip the balance towards coalitions of single or mixed sexes. Additionally, reproductive values can be strongly state-dependent based on future prospects (e.g. age-dependent), and future work could usefully attempt to estimate the most important sources of asymmetries in future prospects for different categories of individuals in each system.

Returning to our case, interactions between immature individuals offer particularly promising avenues for work teasing apart reproductive value considerations along the helper–recipient axis. In this context, we also find it interesting to note that similar effects can take place on the antagonistic side of the spectrum: *Formica aquilonia* ant larvae cannibalize nestmate eggs, and this behaviour is much more common among male than among female larvae [28]. Males are the rare sex in this species [29], and it is thus possible that the rarer-sex effects alone are sufficient to explain why they are the more harmful sex (i.e. contributing less, once we redefine ‘let others live’ as a contribution towards brood survival). *Formica* species also often show split sex ratios (i.e. colonies specializing in either female or male production [30]), which creates similar conditions to the single-sex broods of unmated pine sawflies—but, in the *Formica* case, this can create broods of either sex. As a whole, our models thus point to interesting research avenues for teasing apart various effects favouring more or less cooperative individuals with sexual or other (e.g. age [31]) asymmetries. Whether and when individuals should specifically direct help towards a particular sex even if both sexes are present in the same group is a clear future avenue for work.

## Data Availability

All the code which can be used to reproduce the model is permanently available on Dryad [32]. Supplementary material is available online [33].
